# The Volapük Qur’an: language, scripture, and nineteenth-century German universalist provincialism

**DOI:** 10.1080/17597536.2024.2367355

**Published:** 2024-09-18

**Authors:** Johanna Pink

**Affiliations:** Department of Oriental Studies, University of Freiburg, Freiburg, Germany

**Keywords:** Volapük, Qur’an, Germany (nineteenth-century), Islam, Christianity, international auxiliary languages, constructed languages, Esperanto

## Abstract

This article focuses on the translation of selected segments from the Qur’an into the auxiliary ‘world language’ Volapük that was published by Johann Martin Schleyer, the inventor of the language, in Constance, Germany, in 1890. I investigate the place of this translation within Schleyer’s vision of creating a world language, and use it as a case study through which to explore the ways that literature was framed and recreated within the relatively short-lived endeavour to promote Volapük as *the* new universal language. Analysing the impact that Schleyer’s German Catholic background had on his presentation of the Qur’an, this article sheds light on the provincialism of this specific brand of nineteenth-century European universalism that sparked the construction of Volapük, especially when contrasted with a later project of translating the Qur’an into Esperanto. These findings will allow us to situate the Volapük translation of the Qur’an within the broader field of Qur’an translations that have primarily emerged from language activism, as opposed to a missionary or academic engagement with the Qur’an.

## Introduction

In 1890, in the Southern German city of Konstanz, a booklet comprising a collection of excerpts from the Qur’an into the ‘world language’ Volapük was published. Sold at the moderate price of 45 pfennig, the booklet, which contained no more than fourteen pages of text, was entitled *Sets veütikün ä blefikün mosleminbiba (‘korana’)* or ‘The most important and concise sentences from the Muslim Bible (“Qur’an”)’ (Schleyer 1890). The translator was none other than the Catholic priest Johann Martin Schleyer (1831–1912), the inventor (‘datuvel’) of Volapük. In contrast to most Qur’an translations, Schleyer’s work aimed neither at familiarising non-Muslims with the contents of the Qur’an, nor at helping Muslims understand their faith. Instead, it was intimately related to the efforts made by Schleyer and the Volapükists of his time to develop and popularise the new language, which was only just eleven years old at the time, and to elevate its status to that of a literary language.

This paper investigates the relationship between Schleyer’s vision of creating a world language and his translation of the Qur’an. It uses *Sets veütikün ä blefikün mosleminbiba* as a case study through which to explore the ways in which literature was framed, translated, and reconstructed within the relatively short-lived enterprise that was Volapük. Analysing, from a historical and textual-critical angle, the impact that Schleyer’s German Catholic background had on his presentation of the Qur’an, the article sheds light on the provincialism of the specific nineteenth-century continental European brand of universalism that sparked the construction of Volapük, especially when contrasted with a later project of translating the Qur’an into Esperanto. In my conclusions, I will situate the Volapük translation of the Qur’an within the broader field of Qur’an translations that have primarily emerged, and continue to emerge, from language activism.

## Volapük and its literatures

Volapük literally means ‘world language’, from *vol *= ‘world’ and *pük* = ‘language’. Its inventor, Schleyer, was a Catholic pastor in the small German parish of Litzelstetten near Konstanz in the Southwest of Germany, an occupation that left him ample time for pursuing his two great passions: poetry and the study of languages. He pursued the latter activity in much the same way that he had earlier acquired Latin: through the perusal of grammars and dictionaries. In addition to these interests, he published on just about every conceivable topic, from a cure for diphtheria to the benefits of the patriarchate, and from industrialisation to peace on earth (Sleumer 1914: 9–10). He first came up with the idea for creating a world language in 1878, inspired by the technological progress of the era in the fields of communications and transport and with a vision of a united humanity, and he claims that he was at that time unaware of the many previous attempts to construct similar languages (Sleumer 1914: 20). His aim was to produce a tool that would remove all barriers to communication throughout the world, later captured in Volapük’s slogan ‘Menade bal püki bal’ (‘One language for one mankind’), and the printing press gave him the means to spread his idea in ways that had not been possible to inventors of languages in previous centuries or even shortly before his time.^1^^1^Garvía argues that it was the other way round; that Schleyer was first inspired to invent Volapük and then came up with a problem that this language was supposed to solve (Garvía 2015: 23). But given that Schleyer had previously developed a ‘world alphabet’ that he had proposed to the Universal Postal Union, and that he had mused about the idea of a world language at least a year before coming up with Volapük, it seems more likely that his inspiration was a result of his intensive engagement with this idea (see also Smith 2011: 26–26). The system of Volapük, which is based on monosyllabic roots and affixes, came to him in what he described as a mystical experience in 1879. Unlike nearly all previous constructed languages, with the exception of an obscure immediate predecessor from France (Pirro 1868), it was predominantly an *a posteriori* language, meaning that its vocabulary was derived from existing languages, as opposed to *a priori* languages that are, or claim to be, based on abstract roots or concepts, as was the case with the philosophical languages of the Enlightenment period. Its success paved the way for further *a posteriori* language projects, including Esperanto (Sanders 2020: 23–24). A first draft of a grammar of Volapük was printed in 1879 in the journal *Sionsharfe*, which Schleyer was publishing at the time, and which was usually devoted to Catholic poetry. By spring 1880, a grammar with a word list had appeared in print as an independent book. The roots that formed the basis of Volapük’s linguistic system were mainly derived from English, which Schleyer considered to be ‘the easiest and most widely known’ ‘from among all the languages of educated peoples’, despite its ‘hopelessly confused orthography’ (Schleyer 1880: 3), and to a lesser extent from other West European languages (Garvía 2015: 23). Volapük had a complex but regular grammar and was designed – at least in Schleyer’s mind – to be ‘phonetically easy’; for example, Schleyer tried to avoid the phoneme ‘r’ in order to make the language accessible to Chinese speakers. However, his understanding of phonetics was not based on any linguistic evidence but rather on his own experiences and aesthetic preferences. For example, he was an ardent advocate of umlauts (ä, ö, ü) which, as a consequence, featured heavily in Volapük and are anything but phonetically easy for speakers of many languages.

Nevertheless, within a decade, Volapük had become the most successful constructed auxiliary language to date, and probably the first that was actively practiced by a substantial number of speakers, which made it a turning point in the history of constructed languages. By 1889, more than a thousand diplomas had been awarded to individuals who were deemed proficient enough to teach the language and fourteen Volapük journals were being published. Around 250 Volapük clubs, some of them with several hundred members, had formed. They spread across every continent, but most were located in Europe, especially in the German- and Dutch-speaking countries, and many of the ones outside Europe were mostly composed of German emigrants. This lack of diversity was exacerbated by the fact that the majority of members were educated, Catholic men. Some degree of elitism, at least, was in accordance with Schleyer’s plans for the language, which he saw occupying the same role Latin had held in former centuries, namely, as a medium of communication for educated men (Garvía 2015: 26).

Throughout this period of growth, Schleyer kept a tight rein on the movement, designating himself its ‘supreme leader’ and retaining the right of veto on all decisions concerning the language. This ultimately unsuccessful quest for unity and supreme leadership mirrored the structure of the Catholic church, to which he was utterly devoted (Smith 2011: 26–30; Garvía 2015: 21–33, 50–56; Haupenthal 1982a: 11) and was a part of the reason for Volapük’s decline, which was as fast as its ascent. Amidst polemics against the language from German and French patriots, who considered the idea of a universal language to be antinational, as well as linguists, who claimed that it lacked a soul, or rather, a *geist*, internal conflicts over Volapük’s complexity arose. Schleyer believed that morphological and grammatical complexity were necessary to ensure precision, while others thought that they detracted from the language’s utility. Defections ensued, and the Third Volapük Congress in 1889, led by the Frenchman Auguste Kerckhoffs (1835–1903), brought about a schism. Whereas Kerckhoffs was an ardent champion of using Volapük as an international commercial language and making it as simple and practical as possible in order to achieve that aim, Schleyer wanted the language to be complex enough to express all conceivable nuances and subtleties. Enraged by the decisions made at the Congress in 1889, he parted ways with Kerckhoff and his followers and established a new Volapük Academy (Garvía 2015: 30–32, 45–48, 52).

By 1894, only fifty Volapük clubs were still active and, by 1910, the language was practically dead, with many of its speakers having joined the Esperanto movement (Smith 2011: 30–31; Garvía 2015: 34–50), despite Schleyer’s polemics against this ‘botched-up’ language (Schleyer 1900). A limited revitalisation occurred in the 1920s in the Netherlands (Smith 2011: 30–31; Garvía 2015: 34–50).

During its short heyday, a substantial body of literature in Volapük was produced (Haupenthal 1982b). Much of this comprised grammar and vocabulary books, which Schleyer strove to provide in an enormous range of languages, from Latvian to Nama and Chinese, as well as journals and practical manuals on matters such as conversation and business correspondence. There was only a marginal attempt to produce original literary works in Volapük, such as a small collection of aphorisms by Schleyer (Haupenthal 1982b: 74), but quite a significant number of translations were published. These fulfilled a number of purposes. For example, they served as teaching and learning aids for the new language, demonstrating how Volapük was supposed to be used. They undoubtedly also helped develop its vocabulary. And, maybe most importantly, they provided evidence of the usefulness of the language and its capacity to produce intelligible text beyond the barest everyday needs. For example, there were a number of back-and-forth translations between Volapük and various European languages that were meant to demonstrate that Volapük was capable of expressing meanings with precision (Haupenthal 1982b: 11, 60, 68). These attempts were not only directed outwards but also inwards, towards those segments of the Volapükist community who saw the only purpose of the language in scientific and commercial communication, whose opponents strove to prove them wrong. One of the most avid proponents of Volapük’s literary use, Siegfried Lederer (1861–1911), translated the fairy tales of the Brothers Grimm and Hans Christian Andersen, as well as a Japanese fairy tale from an unnamed source (Garvía 2015: 28, 174, n. 10; Haupenthal 1982b: 3, 25, 45). This reflects a very nineteenth-century approach to the concept of ‘literature’ as a form of cultural expression that is rooted in national traditions. By including translations from Danish and Japanese sources, Lederer presumably aimed to show that Volapük’s potential as a medium of literary expression transcended the boundaries of German folklore. However, most of the authors whose works were either partially or fully translated into Volapük (the former being more common) were more or less contemporaneous.^2^^2^These included the Dutch Multatuli (Eduard Douwes Dekker), the American Edgar Allan Poe, the Pole Henryk Sienkiewicz, the Austrian Moritz Saphir, and the German Theodor Storm. We also find translations of some short texts by slightly less recent authors, generally writing in German, such as Heinrich Heine, Adalbert von Chamisso, and Friedrich Schiller. There are surprisingly few ‘classics’: nothing except for the first canto of Dante’s *Divine Comedy* and some excerpts from Tacitus and Sallustius that spoke to German nationalist sentiments. Besides poetry and novels, they included some pieces of travel and adventure literature, such as the works of Karl May and the Austrian crown prince Rudolf’s ‘Journey to the Orient’. Overall, the sources and source languages were diverse but German literatures played a central role, and there were hardly any attempts to go beyond the European canon, besides Lederer’s foray into Japanese folklore.^3^^3^The only other exception is a translation of an Arabic work that was already available in several European translations, namely, the *Fables of Lokman the Wise*. These were presented in Volapük and nine further (European) languages; for the translator, Volapük seems to have been little more than a new specimen in his collection of languages, and maybe this was not so different when it comes to Schleyer himself. For an overview of Volapük literature and details of all the titles mentioned here, see Haupenthal’s bibliography (Haupenthal 1982b).

Not a single one of these literary translations was undertaken by Schleyer himself, though, and that cannot have been because he shunned the use of his language for such purposes. After all, he valued aesthetics over utility, linguistically speaking (Garvía 2015: 52), and he regularly published Catholic poetry, epics, and dramas in German (Sleumer 1914: 8; 28; Haupenthal 1982b: 70–94). Furthermore, the issues of his journal *Volapükabled* were scattered with his own translations of sentences from classics such as Shakespeare, Cicero, and Goethe, although he never translated longer segments of these works. His predominant interest was his faith, and religious texts were the only genre to which he was willing to devote the necessary time and effort to translate longer chunks of text. The only other members of the Volapük community who seem to have shared his preference were two Jews living on the other side of the Atlantic: in 1889, at the peak of the Volapük movement (Garvía 2015: 47), a translation of the Proverbs of Solomon by the printer Samuel Huebsch was published in New York, and an excerpt from the Talmud in Guadalajara, Mexico, by Joel Elk (Haupenthal 1982b: 63; 103). For Schleyer himself, Catholicism was part and parcel of his project, or rather, his project was part of his Catholicism. As mentioned above, the first draft of his language was published in his journal *Sionsharfe*, which was devoted to Catholic poetry, and the very first sample sentence was ‘With God let us begin all things!’ (*Sionsharfe* 35:1879) The following issue included a segment entitled ‘First Samples of Verse in Our “World Language”’, which starts by affirming that God will help in times of need and continues with the poem: ‘O kim falön Kanóm mekön Sánikün kilgi, Góda kindomi?’ (‘Oh who can make the Holiest Church, God’s Kingdom, fall?’) (*Sionsharfe* 36:1879, 317). A translation of the Magnificat was not long to follow (*Sionsharfe* 40:1879, 350). Once Schleyer published the *Volapükabled* as a separate journal from the *Sionsharfe*, he mostly kept religion out of it, but his grammar books were interspersed with religious sample texts such as the Lord’s Prayer and various psalms (Schleyer 1880: 38; 1887b: 50). And finally, apart from his collection of aphorisms (Schleyer 1888b), the only Volapük texts that he published as independent books or booklets were translations of scripture. Possibly, his motivation to produce these went beyond his own religious inclinations, though. The rise of vernacular languages in Europe, especially in Schleyer’s native language, German, had been closely connected to the printing of Bible translations. For any language to claim the status of a literary language, it needed both ‘high literature’ and scripture. While Volapükists such as Lederer provided the first, Schleyer apparently felt obliged to provide the second; both endeavours happened simultaneously, mostly between 1887 and 1890. During these years, Schleyer published the *First Epistle of John* (1888), two booklets with phrases from the Old and New Testament, respectively (1887, 1889), and, and as a distinct outlier from his general interest in promoting Catholicism, his ‘Qur’an translation’ (first printed in 1890, with a second edition in 1892).

## The Qur’an in Volapük

While Schleyer’s Qur’an translation deviated from his usual focus on Christianity, it fit perfectly into a very peculiar genre of translation that he had created in previous years and that might be dubbed the genre of ‘one hundred most important, meaningful and concise sentences’. He started with aphorisms, which lend themselves to such a structure, but he also imposed this system on the Old and New Testament and eventually also on the Qur’an. Maybe this was because neither Schleyer nor anyone else in the Volapük community had the capacity to translate the entire New Testament or the entire Qur’an, but it is more likely that it was based on Schleyer’s preference for collecting and translating concise statements that ring true (Sleumer 1914: 10), and on his desire, typical of a late nineteenth-century man, to create order. He published a vast number of titles that contained numbers, whether it was ‘three dozen remedies’ or ‘fifty rules of travel’ or ‘the hundred forms of love’ or ‘one hundred reasons to remain a Catholic’ (Haupenthal 1982a: 6; 2002: 22; 1982b: 92). His Biblical translations comprised collections of a hundred verses each, under thematic headers which were arranged alphabetically. For example, in his selection from the Old Testament, Proverbs 13:24 (‘Whoever spares the rod hates his son’^4^^4^Biblical translations in this paper are based on the English Standard Version.) is placed under the header ‘Zùht’, which is how Schleyer spelled the German word *Zucht* (‘discipline’, ‘correction’) in his ‘universal phonetic alphabet’, and it is ranked 99, just before the last item which is titled ‘Zurähtveisung’ (*Zurechtweisung*, meaning ‘rebuke’) – an interesting choice of ‘most meaningful’ topics. Each page of German translation in phonetic spelling is followed by a page of Volapük translation, but the order of items is dictated by the German version; for example, *Zurechtweisung* is titled ‘Gijonam’ in Volapük but is still placed last (Schleyer 1887a: 22–23). For the New Testament, the system is slightly different. For example, Matthew 25:46 (‘And these will go away into eternal punishment, but the righteous into eternal life’) is shortened to ‘Die Gerechten werden eingehen in ewiges Leben’ (‘The righteous will go into eternal life’) and placed under the German-Volapük header ‘Gerechtigkeit (cöd)’ (‘justice’), which is ranked forty-sixth. Then the sentence is given in transliterated Greek, German in standard spelling, German in Schleyer’s phonetic spelling, and Volapük (Schleyer 1889: 15). The inclusion of Greek is linked to Schleyer’s claim, made on the title page, of having translated straight from the Greek *Codex Vaticanus*. He makes no comparable claim for the Old Testament or the Qur’an.

And indeed, while the number of languages that he engaged with ‘somewhat intensively’ is claimed to have totalled fifty-five, or maybe even eighty-eight, and to include a wide range of European, Asian and African languages, Semitic languages are conspicuously absent from the (possibly incomplete) list of languages that he is said to have dabbled in (Sleumer 1914: 6, 9, 13; Haupenthal 1982a: 7, 9).^5^^5^Languages that are mentioned specifically in the biographical sources are Latin, Greek, Russian, English, Italian, Portuguese, Rhaeto-Romance, Swedish, Hungarian, Chinese, Nama, Fang, Asante, Herero, Sámi, Vietnamese, Dayak, Croatian, Malagasy, Gothic, Sanskrit, Slovenian, Thai, Turkish, Sorbian, and Prakrit. And the study of his Qur’an translation provides further evidence for his lack of knowledge of Arabic. When compared to the New Testament, the Qur’an translation lacks both the source text and translations into further languages; it is in Volapük only.^6^^6^Schleyer’s Qur’an translation does not contain any paratexts, nor does he discuss it in the newspapers he published. At the moment, it is impossible to gain access to his diaries, according to an email from Bayerische Staatsbibliothek (5 December 2023). This means that my conclusions are exclusively derived from an analysis of the translation itself. Schleyer had good reason for omitting the German translation because, based on a careful examination of his Qur’an translation, it is clear that he based his Volapük rendering exclusively on the – rather mediocre – German Qur’an translation by Ludwig Ullmann, first published in 1840, reprinted multiple times and the most widely available Qur’an translation in Germany in the late nineteenth century (Ullmann 1865). The use of an existing Qur’an translation by a translator who did not know Arabic was, of course, not unusual. In the nineteenth century, a substantial number of translations into European languages such as Spanish and Italian were based on the French translations by Étienne de Savary and Albert de Kazimirski, for example. Schleyer, however, did not credit Ullmann, despite the fact that he translated his text word for word (and, incidentally, the fact that this was even possible without any shifts in syntax tells us a lot about the inherent Germanness of Volapük). Schleyer even copied the word formation of Ullmann’s German usage. For example, in Q 4:171, Jesus is described as being God’s word which He cast into (*alqā*) Mary. Ullmann renders this verb as ‘übertragen’, a word that is composed of the prefix ‘über’ (‘over’) and the root ‘tragen’ (‘to carry’) and means ‘to transfer’. Schleyer renders it as ‘ovepolön’, composed of the prefix ‘ove’ (‘over’) and the root ‘polön’ (‘to carry, to transport’), constructing a compound verb in Volapük that mirrors Ullmann’s word choice where the stem would actually completely suffice to convey the intended meaning.

The two examples in Table 1 and Table 2 further demonstrate Schleyer’s complete reliance on Ullmann.^7^^7^English quotations from the Qur’an, unless otherwise noted, are based on the translation by Alan Jones that has the advantage, in the present context, of staying relatively close to the source text and bearing some similarity to Schleyer’s laconic style (Jones 2007).^,8^^8^My translations from Volapük are based on an English textbook, dictionary, and grammar that were published around the time Schleyer wrote his Qur’an translation, as well as the fourth edition of Schleyer’s own dictionary which was published in 1888 (Sprague 1888; Wood 1889; Post 1890; Schleyer 1888a).

**Table 1.** Schleyer’s and Ullmann’s translations of Q 25:71.

Original	English translation
وَمَن تَابَ وَعَمِلَ صَالِحًا فَإِنَّهُ يَتُوبُ إِلَى اللَّهِ مَتَابًا	Those who repent and act righteously turn to God in repentance.^7^
Aikel pönitom, e dunom vobädis gudik: ota levotäl al Godi binom pöcedöl, as känüdik (Schleyer 1890: 9).	Whoever repents, and does good works: the same one’s conversion to God is to be judged as sincere.^8^
Wer bereut und gute Werke verrichtet, dessen Bekehrung zu Gott ist als eine aufrichtige zu halten (Ullmann 1865: 308).	Whosoever repents and does good works, his conversion to God is to be considered a sincere one.

**Table 2.** Schleyer’s and Ullmann’s translations of Q 13:2.

Original	English translation
اللَّهُ الَّذِي رَفَعَ السَّمَاوَاتِ بِغَيْرِ عَمَدٍ تَرَوْنَهَا ثُمَّ اسْتَوَىٰ عَلَى الْعَرْشِ وَسَخَّرَ الشَّمْسَ وَالْقَمَرَ كُلٌّ يَجْرِي لِأَجَلٍ مُّسَمًّى يُدَبِّرُ الْأَمْرَ يُفَصِّلُ الْآيَاتِ لَعَلَّكُم بِلِقَاءِ رَبِّكُمْ تُوقِنُونَ	It is God who raised up the heavens without visible pillars; then He set himself on the throne and subjected the sun and the moon to His command, **each running for a stated term. He directs the affair**, making His signs distinct, so that you (pl.) may be convinced that you will meet your Lord.
Silakòps, valik ailaboms külifümik okas. Om (GOD) leodom dinis alik. (Schleyer 1890, 14)	The celestial bodies, all are having their definite courses. He (GOD) orders all things.
Alle Himmelskörper haben ihren bestimmten Lauf. Er ordnet alle Dinge (Ullmann 1865: 201).	All celestial bodies are having their definite course. He orders all things.

As we can see from the second example, Schleyer did not choose entire Qur’anic verses, but took bits and pieces from them, and even cut across verses. He sometimes did the same with Biblical verses, but in the case of the Qur’an this was exacerbated by the fact that the concept of the division of suras into ‘verses’ (*āyāt*) clearly eluded Schleyer. He instead mostly relied on Ullmann’s punctuation, which is alien to the Qur’anic source text. Furthermore, Ullmann’s translation, like all nineteenth-century Qur’an translations into Western European languages and many contemporaneous Arabic copies of the Qur’an, did not contain verse markers, let alone verse numbers. For Schleyer, each sura was one long continuous passage consisting of German sentences from which he picked some that suited his purpose. He also copied some of Ullmann’s mistakes, which demonstrates that he was not even using the Arabic text as a backup. For example, according to Q 57:27, God placed ‘compassion and mercy’ (*raʾfatan wa-raḥmatan*) in the hearts of Jesus’ followers. Ullmann, however, mistranslates this as ‘Frömmigkeit und Erbarmen’ (‘piety and mercy’), and so does Schleyer (‘relöfi e misaladi’) (Schleyer 1890: 16; Ullmann 1865: 474).

Using Ullmann’s translation, it would have been hard for Schleyer to convey the rhetoric and impact of the Qur’anic text, but this was clearly not something he set out to do or designed his language to achieve. His focus was exclusively on conveying meaning and on the most efficient way of transmitting its essence, as opposed to its nuances. He was not interested in narrative, in emotion, or in aesthetics. While a focus on content over rhetoric is not uncommon at all in Qur’an translation (Pink 2019), this is usually due to either to a grammatical approach, on the part of philologists, or to a Muslim translator’s idea of the Arabic Qur’an as an inimitable text. However, in Schleyer’s case, it mainly reflects his entirely mechanical notion of language as a tool, the correct use of which can be learned through studying a manual (Garvía 2015: 55).

For example, in his section on ‘hell’ (‘höl’), Schleyer includes a translation of Q 82:13–14:

                                                                                  إِنَّ ٱلْأَبْرَارَ لَفِى نَعِيمٍۢوَإِنَّ ٱلْفُجَّارَ لَفِى جَحِيمٍۢ

*Inna l-abrāra la-fī naʿīm*

*wa-inna l-fujjāra la-fī jaḥīm*

Due to its rhythm and rhyme, this segment is highly expressive in the original Arabic. Schleyer, however, limits himself to conveying the bare meaning: ‘Cödikans okömoms in paladi vonik; abu klimidunels in höli’ (‘The just people will come into a delightful paradise; the criminals, on the other hand, into hell’) (Schleyer 1890: 7). In all fairness, most translators fail to capture the rhyme in this segment, but some make at least an effort to convey the emphasis it carries and the rhythm and parallelism between the two verses. This is not something that is impossible to achieve even in an auxiliary language, as can be seen in Abdul Hadi Italo Chiussi’s Esperanto translation (Chiussi 1970: 625), first published in 1969, about which more will be said below:

Vere la justuloj certe estos en feliĉo,

Kaj la pekuloj certe estos en la Infero.

In contrast to Chiussi’s work, all the segments Schleyer translated are consistently very brief, free of flavour and detail, and without an aesthetic impact. This becomes all the more obvious since their core meaning is typically less than surprising; the reader would hardly have expected the righteous people not to enter Paradise.

Why did Schleyer translate segments from the Qur’an at all, then? What meaning did he hope to bring across? If we take a closer look at his choice of topics and segments, and his strategic omissions, we gain an impression of the deep ambivalence between the universalism inherent in his linguistic project and his unshakeable, particularistic belief in the superiority of Christianity.

## Volapük universalism and the supremacy of Christianity

One obvious reason for producing this ‘Qur’an translation’ was Schleyer’s claim for the universality of Volapük, which clashed with the general Eurocentrism of published Volapük literature. After translations of the Jewish and Christian scriptures had been produced in the late 1880s, the third major Abrahamic religion was a lacuna and Schleyer took it upon himself to fill this gap. There is no indication that Schleyer’s frame of reference, despite all his studies of African and Asian languages, went beyond the Abrahamic religions, but he does seem to have had the intuition that, for Volapük to claim the status of a universal language, it needed to embrace more than the Jewish and Christian scriptures. Schleyer might also have been motivated to demonstrate Volapük’s claim to universality because he had been deeply affected by the *kulturkampf*, the Prussian state’s persecution of the Catholic church during the 1870s. Schleyer himself had been denounced by members of his parish and confined in the fortress of Rastatt for four months in 1875. Schleyer’s Catholicism was also held against his invented language by some of its opponents (Garvía 2015: 53–55), which might have provided an additional impetus to make the language appear more universal.

At the same time, the Qur’an was the book of a false prophet to Schleyer, devoted Catholic that he was. He felt no particular inclination to represent it truthfully and it is clear that his selection of segments from the Qur’an was governed by a theological agenda. For example, rather than choosing precisely one hundred sentences, as he did with the Old and New Testament, Schleyer instead picked one hundred topics and then placed anything between one and twenty-two sentences or segments under these headers. He treated some of these in a rather lacklustre manner and probably only included them to reach the required number of one hundred topics; otherwise, it would be hard to explain the high number of vague or redundant headers. For example, he has separate sections for ‘the grace of God’, ‘the mercy of God’ and ‘God’s love’, none of which contain more than a sentence, and the difference between them does not seem to go beyond semantics. At the other end of the spectrum is the header ‘God’, with twenty-two sentences. The second-most extensive topic is ‘Jesus’. In addition, he has a section on ‘Christians’ but not on ‘Jews’, even though Jews feature prominently in the Qur’an. Judging from the fact that Schleyer even covers ‘idolatry’ and is thus entirely aware of the fact that the Qur’an discusses non-Muslims other than Christians, this omission appears to be a deliberate choice.

Schleyer strategically chose the sentences he translated, which constitute only a fraction of the Qur’anic text, so that statements he would have found problematic from a Christian point of view were consistently excluded. For example, his comparatively long section on God starts with the uncontroversial snippet ‘Geisubimik aibinom GOD, el Reg, el Velat!’ (‘Most exalted is GOD, the King, the Truth!’) (Schleyer 1890: 5). This is the beginning of Q 23:116. The next part of the verse says ‘There is no god but Him’, emphasising Islam’s undivided monotheism in what was often understood as an implicit attack against the doctrine of the Trinity. This statement was omitted by Schleyer. That such choices are no coincidence becomes evident when examining his translations of verses that are explicitly related to Christian beliefs and practices.

For example, Q 4:171 starts with an exhortation directed at the ‘People of the Book’, a label that the Qur’an uses to describe the Jews and Christians. The verse continues with a statement on Jesus and ends with a polemic against the Christian doctrine of Trinity:

O People of the Scripture, do not go beyond the bounds in your religion. Do not say anything about God. *al-Masīḥ*, Jesus, the son of Mary, is truly God’s messenger, and His word, which He cast into Mary, and a spirit from Him. So believe in God and His messengers and do not say ‘Three’. Desist. [That is] better for you. God is one God. Glory be to him – that He should have a son […]

Schleyer only translated the middle part of this verse: ‘Truly! The Messiah Jesus, son of Mary, is a messenger of God, and His Word, which He has transferred unto Mary, and His Spirit’. (‘Velatö! Mesial Yesus, son ji*el*a Maria, aibinom legat’al Goda, ed Oma Vöd, keli elove*pol*om in ji*el*i Maria, e Tikäl Oma’) (Schleyer 1890: 16). Read this way, the Qur’anic segment appears to be a confirmation of Jesus’ status, rather than a critique of Christian beliefs.

Schleyer dealt with Q 57:27 in a similar fashion:

Then We caused Our messengers to follow in their footsteps. We caused Jesus, the son of Mary, to follow and We gave him the Gospel; and We placed compassion and mercy in the hearts of those who followed him. But monasticism they invented. We did not prescribe it for them [but it arose] through desire for God’s satisfaction; and they did not observe it as they should have done. So We gave those of them who believed their wage; but many of them are profligates.

Here, Schleyer only translated the first part: ‘We caused to follow [after] them (i.e. the messengers) Jesus, the son of Mary, and him we gave the Gospel, and placed in the heart of those who followed him piety and mercy’ (‘Emekobs sukön omes (legat’ales) Yesus’i, soni jiela Maria, ed Ome ägivobs eli gospeli, ed äseitobs in ladi utas, kel esukoms Ome, relöfi e misaladi’) (Schleyer 1890: 16). The critique directed against monasticism and ‘many’ of the Christians in the second half of the verse is missing.

The omission of the beginning and end of Q 4:171 could theoretically be justified by Schleyer’s method of selecting only single sentences from Ullmann’s text, rather than verses or thematic units, since Ullmann had a full stop before and after the quotation selected by Schleyer. But, in the case of Q 57:27 Schleyer cut the quotation short after a semicolon, and in the following example, Q 41:33, he even left out part of an ongoing sentence. Ullmann renders the verse as follows:

Wer führt wohl eine schönere Sprache als Der welcher die Menschen zu Gott einladet und rechtschaffen handelt und sagt: Ich bin Moslem? (Ullmann 1865: 413)

(Who might use a more beautiful language than the one who invites humans to God and acts righteously and says: I am a Muslim?)

In Schleyer’s Volapük version, the verse reads: ‘Who could use a more beautiful language than the one who invites humans to God and acts with integrity?’ (‘Kim ba gebom püki jönikum, ka ut, kel vüdom menis al Godi, e dunom rito?’) (Schleyer 1890: 13). The crucial component at the end that turns this rhetorical question into a reference to Muslims – if this is how one chooses to translate the Arabic term *muslim –* is missing, and there can be no doubt that this was a conscious, strategic omission. Schleyer wanted to translate the Qur’an on the basis that it is a scripture, to enhance the universality of Volapük’s claim, but at the same time he was loath to acknowledge the Qur’an’s polemics against Christian beliefs or include anything that could be understood as an affirmation of the veracity, let alone superiority, of Islam.

A comparison with Chiussi’s Esperanto translation of the Qur’an, published approximately eighty years later, throws Schleyer’s brand of particularism into sharp relief. Chiussi was a world-renowned Esperanto activist (Astori 2019b: 129) who developed some interest in Islam after meeting representatives of the Ahmadiyya Muslim Jamaat (AMJ) in Germany and subsequently translated the entire Qur’an, producing what a contemporary Esperanto writer counted among the ‘core works’ (*la kernaj verkoj*) of translated Esperanto literature (Astori 2019a: 85). Chiussi embraced Islam as a result of this venture (Astori 2019a: 87–88). The AMJ had emerged as a missionary movement in British India in the late nineteenth century and had been pioneers in the attempt to spread Islam through Qur’an translations. The AMJ’s efforts later, in 1989/1990, culminated in a campaign to produce translations into 101 languages of a book of excerpts from the Qur’an in celebration of the movement’s centenary, including such unlikely candidates as Yiddish, Batak, Xhosa, and Welsh (‘Selected Verses of the Holy Quran with Translation’ n.d.). For Chiussi, the universalism of Islam (understood according to Ahmadiyya teachings) and the universalism of Esperanto went hand in hand, and this was what motivated him to translate the entire Qur’an: ‘I was particularly attracted by the idea of transferring the message of peace (Islam also means peace) into the “language of peace”’^9^^9^‘Mi estis aparte altirita de la ideo translokigi la mesaĝon de paco (Islamo ankaŭ signifas acon) en la “lingvon de la paco”.’ (Astori 2019a: 87).

Even without any knowledge of Esperanto whatsoever, it is easy to see how this differs to Schleyer’s project. Consider, for example, Chiussi’s rendition of Q 94 (Chiussi 1970: 641):

Ĉu Ni ne dislarĝigis por vi vian bruston,

kaj forprenis de vi vian ŝarĝon,

kiu ŝarĝis vian dorson?

Ĉu Ni ne pliigis por vi vian famon?

Vere post la malfeliĉo venas la faciligo;

post la malfeliĉo venas la faciligo,

kiam do vi estas senŝarĝita, vi fervoru,

kaj dediĉu vin al via Sinjoro.

For Chiussi, the Qur’an is an expressive text that includes rhyme, rhythm, and parallelisms, all of which underline its emphatic appeal to its audience, and he had no difficulties bringing this across in Esperanto. Thus, the limits of Schleyer’s Qur’an translation in terms of its extent, precision, and style are not a result of the fact that Volapük was a constructed language. Rather, they betray an ultimately very provincial brand of cosmopolitanism that is fairly typical of the times. It is reminiscent of the worldview of Karl May, a hugely successful, contemporaneous German author of adventure novels, some of whose stories were translated into Volapük. Karl May had his alter ego travel the world as ‘Old Shatterhand’ and ‘Kara Ben Nemsi’, blend in with the natives, speak their language flawlessly, and demonstrate his German superiority in every way, from playing the organ to the killing of enemies. He would ultimately inevitably convince even the noblest of the savages to embrace Christianity (Ferens 2008). In a similar fashion, Schleyer purported to have mastery of dozens of languages from around the world and, on this basis, to have created a truly universal medium of communication that could serve all peoples. But, at the same time, his language explicitly targeted only the educated elites (*Gebildete*), and his view of who they were and what they believed in was very narrow. His project was deeply entangled in a provincial German, Catholic worldview, the limitations of which he was probably completely unaware. He had a nineteenth-century mentality of limitless faith in human progress, believing that universal communication could be engineered and that the scriptures of world religions could be categorised into chunks of meaning presented in sets of a hundred. But the meaning that he was purporting to derive from the Qur’an was predetermined by his own convictions.

Schleyer’s version of the ‘Mosleminbib’ was clearly not distributed widely, and today only a handful of libraries in Germany, the Netherlands, and Switzerland hold copies. Yet its position at the nexus of language, literature, and religion raises questions that continue to be relevant until today: Why are specific languages chosen as target languages for Qur’an translations? How does the act of translation change their prestige, and what impact does it have on the status of the Qur’an?

## Conclusion: the Qur’an and language activism

That Biblical translations have played a role not just in preaching and proselytising, but also in the production and standardisation of literary languages, in elevating their status and defining their vocabulary, has been shown for many different contexts (see, e.g. Naudé 2005). In the study of Qur’an translations, such questions have for a long time been pushed to the margins by debates about the permissibility of translating the Qur’an, the correct way of doing so, and the achievements, or lack thereof, of individual translations in capturing its meaning, with a heavy focus on English (Pink 2015). This is only slowly starting to change. The focus in the more recent body of research on the historical context in which modern Qur’an translations emerged is typically on languages that ended up gaining the status of a ‘national language’ (Lukman 2022; Wilson 2014; Gunasti 2019; Henning 2015). However, the use of the Qur’an to enhance the status of a marginalised language is nowadays more common than ever, and many of these projects are strongly motivated by language activism, just as Schleyer’s and Chiussi’s were. For both of them, it would have been entirely clear that there was not a single person in their potential readership who would not already have had access to the Qur’an’s meaning in some other language, a language in which they were literate and had received an education. But it was because Schleyer and Chiussi wanted to raise their language to the status of those other languages that they felt compelled to translate the Qur’an, or at least a small part of it. Looking at such efforts might also help us take a different perspective on many recent translation projects.

For example, in 1982 the Mauritanian intellectual Oumar Bâ (1917–1998) published a Qur’an translation into French and Poulaar, his native language, which had ended up being a minority language in Mauritania, Senegal, and several other postcolonial African nation-states (Bâ 1982). It was the culmination of decades of effort to save his language and his home region of Futa Toro, which had been arbitrarily divided between Mauritania and Senegal, from falling into oblivion (Bâ 1972; Kane 1998). The potential readership of his Poulaar translation was small, if there was any at all, because graduates of traditional Islamic educational institutions would have learned to read the Qur’an in Arabic whereas graduates of ‘modern’, secular schools would have been more familiar with reading French than Poulaar, a language that had no standardised spelling at the time. Bâ’s Qur’an translation aimed to preserve his region’s linguistic heritage and to support the efforts of language activists at obtaining a recognised status for Poulaar (Hames 2017).

In a similar fashion, the King Fahd Qur’an Printing Complex, a Saudi government institution in Medina, published a Romani translation of the last part of the Qur’an in 2002 (*Fatiha em o Džuzu Amme. Em leskoro iraniba ki Romani čhib* 1422). The translator, Muharem Serbezovsky (b. 1950), is a Muslim Roma singer and Romani activist who claimed that his next project would be a Romani Bible translation (Silverman 2012: 304–305, nn. 18, passim; Radio Free Europe 2005). Romani, like Poulaar, is spoken across many countries and is a minority language in all of them. Educated Roma are typically literate in the dominant language of the country they live in, such as Bosnian. On Serbezovsky’s part, the desire to translate the Qur’an was clearly born out of language activism. Conversely, the King Fahd Complex allowed Serbezovsky, who, as a singer without religious training, did not meet many of the requirements they typically expect their translators to fulfil, to produce this work for them because they, too, had something to gain from it: their ability to produce Qur’an translations into an ever-growing number of languages, including rare or marginalised ones, demonstrates the universality of the Qur’an’s message and highlights Saudi Arabia’s commitment to spreading Islam worldwide. Similar dynamics may be observed when it comes to many other institutions that systematically publish Qur’an translations. For example, the Turkish Directorate of Religious Affairs has published translations into languages such as Chewa and Shona under the slogan ‘No language without a Qur’an’ (*Hiçbir Lisan Kuransız Kalmasın*; Şahin 2017), and the Indonesian Ministry of Religious Affairs, after decades of promoting the Indonesian language only, started a programme to translate the Qur’an into a large number of regional languages, despite the fact that literate speakers of these languages are usually more familiar with reading Indonesian (Mardhatillah 2024).

Translations born out of language activism fall outside of frameworks that categorise translations of Islamic text by their intended target readership and communicative purpose, for example distinguishing between academic, polemical, and pious works, or between translations that centre pedagogy, curiosity, and meaning, respectively (Rippin 2014). In translations born out of language activism, the translation is not primarily produced in order to convey meaning; rather, its production is a performative act that implies a claim about the target language, the translated text, and possibly also the publisher of the translation. It enhances the status of the target language, turning it into a literary language that has its own scriptural translation. It elevates the prestige of the institution that publishes the translation by demonstrating its willingness and ability to reach a global readership. Furthermore, it underlines the universality of the Qur’an’s message. In some cases, it may even serve the purpose of claiming a language for Islam, as was clearly one of the motives behind the production of the first Afrikaans Qur’an translation, about twenty-five years after the Bible had been translated into Afrikaans (Baker 1961). This is where the parallels to Schleyer’s translation end, however. The idiosyncrasy of Schleyer’s Qur’an translation, which clearly sets it apart from later, Muslim-induced projects (including Chiussi’s Esperanto translation), lies in the fact that he wanted to enhance his own prestige and that of his language, but he did not want to risk promoting the Qur’an’s message or, more generally, the truth claims of Islam. And he did not shy away from manipulating the Qur’anic text to achieve that purpose – a text that he introduced in the title as a ‘Muslim Bible’, thereby fitting it into a Christian frame of reference, even though nothing in the language he was inventing prevented him from calling it a ‘Koran’.

This reflects an attitude that I label ‘universalist provincialism’, which permeates Schleyer’s work as well as that of many other nineteenth-century Germans, such as the above-mentioned Karl May. Men like May and Schleyer strove to be cosmopolitan, to educate their readers about the world and, not least, to impress them with their knowledge of it, but at the same time they looked at the entire world through the lens of their own provincial perspective. Schleyer’s approach cannot even truly be called ‘Eurocentric’; it was firmly rooted in his South German upbringing and environment. His language was supposed to be a world language, but his ‘meta-language’ (Garvía 2015: 52) was Catholicism, along with the authority structures inherent in the Catholic church which he had learned to defend during the Prussian *kulturkampf. Sets veütikün ä blefikün mosleminbiba* constitutes an attempt to broaden the literary universe of Volapük without ever challenging its author’s beliefs. Schleyer never wanted his readers to become immersed in seventh-century Arabia, where the Qur’an originated. Instead, he took the Qur’an to Litzelstetten.
